# Ferroptosis-Related Gene Signature Predicts the Prognosis of Skin Cutaneous Melanoma and Response to Immunotherapy

**DOI:** 10.3389/fgene.2021.758981

**Published:** 2021-11-03

**Authors:** Ziqian Xu, Yihui Xie, Yaqi Mao, Juntao Huang, Xingyu Mei, Jun Song, Yue Sun, Zhixian Yao, Weimin Shi

**Affiliations:** ^1^ Department of Dermatology, Shanghai General Hospital, Shanghai Jiao Tong University School of Medicine, Shanghai, China; ^2^ Department of Otolaryngology Head and Neck Surgery, Ningbo Medical Center (Ningbo Lihuili Hospital), The Affiliated Lihuili Hospital of Ningbo University, Ningbo, China; ^3^ Department of Urology, Shanghai General Hospital, Shanghai Jiao Tong University School of Medicine, Shanghai, China

**Keywords:** melanoma, ferroptosis, signature, prognosis, risk score

## Abstract

Ferroptosis is a non-apoptotic regulated cell death process, and much research has indicated that ferroptosis can induce the non-apoptotic death of tumor cells. Ferroptosis-related genes are expected to become a biological target for cancer treatment. However, the regulation of ferroptosis-related genes in skin cutaneous melanoma (SKCM) has not been well studied. In the present study, we conducted a systematic analysis of SKCM based on RNA sequencing data and clinical data obtained from The Cancer Genome Atlas (TCGA) database and the FerrD database. SKCM patients from the GSE78220 and MSKCC cohorts were used for external validation. Applying consensus clustering on RNA sequencing data from TCGA the generated ferroptosis subclasses of SKCM, which were analyzed based on the set of differentially expressed ferroptosis-related genes. Then, a least absolute shrinkage and selection operator (LASSO)-Cox regression was used to construct an eight gene survival-related linear signature. The median cut-off risk score was used to divide patients into high- and low-risk groups. The time-dependent receiver operating characteristic curve was used to examine the predictive power of the model. The areas under the curve of the signature at 1, 3, and 5 years were 0.673, 0.716, and 0.746, respectively. Kaplan-Meier survival analysis showed that the prognosis of high-risk patients was worse than that of low-risk patients. Univariate and multivariate Cox regression analyses showed that the risk signature was a robust independent prognostic indicator. By incorporating risk scores with tumor staging, a nomogram was constructed to predict prognostic outcomes for SKCM patients. In addition, the immunological analysis showed different immune cell infiltration patterns. Programmed-death-1 (PD-1) immunotherapy showed more significant benefits in the low-risk group than in the high-risk group. In summary, a model based on ferroptosis-related genes can predict the prognosis of SKCM and could have a potential role in guiding targeted therapy of SKCM.

## Introduction

Human skin cutaneous melanoma (SKCM) is a highly malignant tumor derived from melanocytes, that is prone to occur in adults. The mortality rate of melanoma is up to 75% (Davis et al., 2019). Early-stage, localized melanoma can be curable if appropriate and sufficient treatment is administered (Coit et al., 2013) ; however, the tumor tends to metastasize and spread to other parts of the body (Aris and Barrio, 2015), once metastasized, the 5-years survival rate decreases to only 15% (Coit et al., 2013). SKCM is now the third most commonly diagnosed cancer in the United States, with an estimated 192,000 new cases in 2019. The incidence is six times higher than 40 years ago (Welch et al., 2021). Surgical resection is associated with a satisfactory prognosis for melanoma in the early stages, while treatment of metastatic melanoma mainly relies on immunotherapy (Leonardi et al., 2020). Many studies have explored the relationship between cancer cells, the tumor microenvironment (TME), and the immune system. However, not all treatments that block immune suppression control points are effective for all patients (Rodríguez-Cerdeira et al., 2017). Therefore, identifying robust predictive biomarkers for both clinical prognosis and treatment response is essential.

In recent years, tumor ferroptosis has gathered much interest. Iron is involved in many biochemical processes in the human body, including oxygen transport, various biosyntheses, and the electron transport chain (as a cofactor), playing crucial roles in cell survival (Bogdan et al., 2016). In mitochondrial oxidative phosphorylation, cells produce reactive oxygen species (ROS) while generating ATP. Excessive ROS levels will lead to oxidative stress, directly or indirectly damaging macromolecules, such as proteins, nucleic acids, and lipids, leading to cell damage or death (Yu et al., 2017). Ferroptosis is an iron-dependent form of programmed cell death, which differs from apoptosis, cell necrosis, and autophagy.

The mechanism of ferroptosis is based on influencing iron metabolism in cells, resulting in intracellular ROS production and excessive oxidation of polyunsaturated fatty acids (Dixon et al., 2012; Battaglia et al., 2020). Ferroptosis is mainly regulated by system X_C_^−^ and glutathione peroxidase 4(GPX4). System X_C_^−^ is a Na^+^-dependent cysteine—glutamic acid exchange transporter in the membrane, which completes the intracellular and extracellular glutamate—cysteine exchange (Sato et al., 1999; Bridges et al., 2012). Cell uptake of cysteine is a crucial step in glutathione (GSH) synthesis, and the generation and maintenance of GSH is the key to protecting cells from ROS damage (Yu et al., 2017). GPX4 is an enzyme that decomposes H_2_O_2_ and organic peroxides into water or the corresponding alcohols, and GSH is an indispensable cofactor in its activation (Ursini et al., 1995). The ferroptosis inducers, erastin and buthionine sulfoximine, reduce the activity of GPX4 and increase the level of ROS in the cytoplasm and lipid (Yang et al., 2014), leading to cell ferroptosis. Ferroptosis is considered as an adaptive process to eliminate malignant cells damaged by nutrient deficiency, infection, or other stress from the body (Mou et al., 2019). A number of studies have demonstrated that ferroptosis plays a role in ischemia-reperfusion injury, cancer, and other diseases. At present, sorafenib and other ferroptosis inducers are used for the treatment of cancer in clinical practice (Fearnhead et al., 2017).

Non-cellular components in the TME may reprogram tumor initiation and invasiveness, but the relationship between iron metabolism and TME is still unclear (Sacco et al., 2021). Tumor-associated macrophages loaded with iron can promote the production of ROS and pro-inflammatory cytokines (tumor necrosis factor-α and interleukin-6), thereby inducing tumor cell death in lung cancer (Costa da Silva et al., 2017). Moreover, new evidence suggests that immune checkpoint blockade decreases tumor growth in a ferroptosis-dependent manner in animal models (Tang et al., 2020), which is considered to be related to antitumor immunity. In addition, the immunotherapy-activated CD8^+^ T lymphocytes induce ferroptosis in cancer cells by downregulating genes (*SLC7A11* and *SLC3A2*) that encode two subunits of the X_C_^−^ system, and the molecular basis behind this phenomenon may be related to interferon (IFN)-γ-mediated transcriptional repression of *SLC7A11* and *SLC3A2* (Wang et al., 2019). Consequently, identifying biomarkers of iron metabolism within the TME may aid the development of effective cancer treatment strategies.

Focusing on melanoma, many studies have found ferroptosis regulators, as well as traditional ferroptosis-inducing agents. It was found that miR-9 reduced erastin- and RSL3-induced ferroptosis in melanoma cells, and knockout of miR-9 could cause ferroptosis in melanoma cells (Zhang et al., 2018). In addition, the inactivation of miR-137 enhances the anti-melanoma activity of erastin by increasing ferroptosis (Luo et al., 2018). However, it is still unknown whether ferroptosis-related genes are related to the prognosis of SKCM patients.

In the present study, we conducted a comprehensive analysis based on transcript and clinical data obtained from The Cancer Genome Atlas (TCGA) and the FerrD databases. We constructed a predictive model on account of eight ferroptosis-related genes. The model can be regarded as an independent predictor of overall survival (OS) of SKCM. A nomogram was established to further explore the prognosis of SKCM based on risk score and tumor stage. Furthermore, we analyzed the mutational differences of ferroptosis-related genes in high-risk and low-risk groups and the associations between the ferroptosis-related risk score and immune cell infiltration patterns and immunotherapy. External validation cohorts were used to verify the ferroptosis-related risk score predicting the response to immunotherapy of the two subgroups.

## Materials and Methods

### Data Collection

The RNA-sequencing (RNA-Seq) and genomic data for SKCM were downloaded from UCSC Xena (http: //xenabrowser.net/), and the clinical data were downloaded via the R package “TCGAbiolinks”. The samples were screened to retain those, including survival status and survival time. A total of 457 samples were obtained. Ferroptosis-related genes were downloaded from the FerrDb database (www.zhounan.org/ferrdb/operations/download.html) and published literature (Liang et al., 2020) and merged. Thus, 268 ferroptosis-related genes were obtained in the analysis (Supplementary Table S1), from which an expression matrix of the ferroptosis-related genes in the SKCM RNA-Seq data was obtained as a candidate gene set expression matrix.

Additionally, the gene expression profile and clinical data of the two independent cohorts was obtained, GSE78220 (Snyder et al., 2014), from the GEO database (https://www.ncbi.nlm.nih.gov/geo/) and MSKCC cohorts (Newman et al., 2015), from cbioportal (http://www.cbioportal.org/study/summary?id=skcm_mskcc_2014). These two independent cohorts were used as the external validation cohorts.

### SKCM Subclass Identification

The R package “ConsensusClusterPlus” was used to apply consensus clustering analysis to the candidate gene set and to classify results by dividing the samples into two clusters. Survival analysis showed that the prognosis based on the two clusters was significantly different (*p* < 0.001). The differences in ferroptosis-related genes between the two clusters were analyzed using R package “DESeq2” (*p* < 0.05 and |logFC| > 0.5). There were 116 differentially expressed genes (DEGs) identified as a new candidate gene set, containing 63 downregulated genes and 53 upregulated genes. Subsequently, the DEGs were annotated by gene ontology (GO) and using Kyoto Encyclopedia of Genes and Genomes (KEGG) data in R package “clusterProfiler”. Finally, univariate cox regression analysis was performed on the 116 DEGs (*p* ≤ 0.01), for which a total of 28 prognostic-related genes were identified for further analysis.

### Construction and Validation of a Ferroptosis-Related Risk Signature

Using the R package “survival”, univariate Cox regression analysis was performed on the prognostic-related genes, where *p* ≤ 0.001 was regarded as statistically significant. The least absolute shrinkage and selection operator (LASSO) method was used to eliminate overfitting with the R package “glmnet”. Then, multivariate Cox regression was used to select the independent prognostic factors. Meanwhile, the correlation coefficients of these genes were calculated. Using the these genes and corresponding correlation coefficients, a prognosis-related model was constructed. Then, the samples were divided into high-risk and low-risk groups by the median of the risk scores. The receiver operating characteristic (ROC) curve was used to evaluate the survival prediction ability of the model, and the Kaplan–Meier (K-M) method was used to analyze the survival difference between the two risk subgroups. Next, a nomogram was constructed based on the prognostic signature using R package “RMS”, and a calibration plot was drawn to evaluate the consistency between the prognostic model’s actual and predicted survival rates.

### Analysis of Somatic Mutation and Immunotherapy Differences Between High- and Low-Risk Groups

“CIBERSORT” (Wilkerson and Hayes, 2010) in R software was used to analyze the differences in immune cells infiltration between high- and low-risk groups. Meanwhile, the correlation between immune cells and risk score was analyzed. Concomitantly, the difference in response to PD-1 immunotherapy between high- and low-risk groups was verified in GSE78220 and MSKCC cohorts. The mutation differences of ferroptosis-related genes in high- and low-risk groups were analyzed using the R package “maftools”.

### Statistical Analysis

Student’s *t*-tests and Wilcoxon rank-sum tests were used to compare the differences between the high- and low-risk groups. The Kruskal-Wallis test was used for comparisons of prognoses between groups. The K-M method was used to generate survival curves for the subgroups in each data set. The log-rank test determines the statistical significance of differences. Univariate and multivariate Cox regression analyses were applied to define independent prognostic-related factors. The ROC curve was visualized using the R package “pROC”, and the areas under the curve (AUC) and confidence intervals were calculated to evaluate the model’s accuracy in predicting prognosis. All statistical analyzses were performed using SPSS (version 23.0) and R software (version 3.6.1). For each analysis, statistical significance was set at *p* < 0.05.

## Results

### Identifying Two Subtypes and Their Distinct Ferroptosis Patterns

**FIGURE 1:**
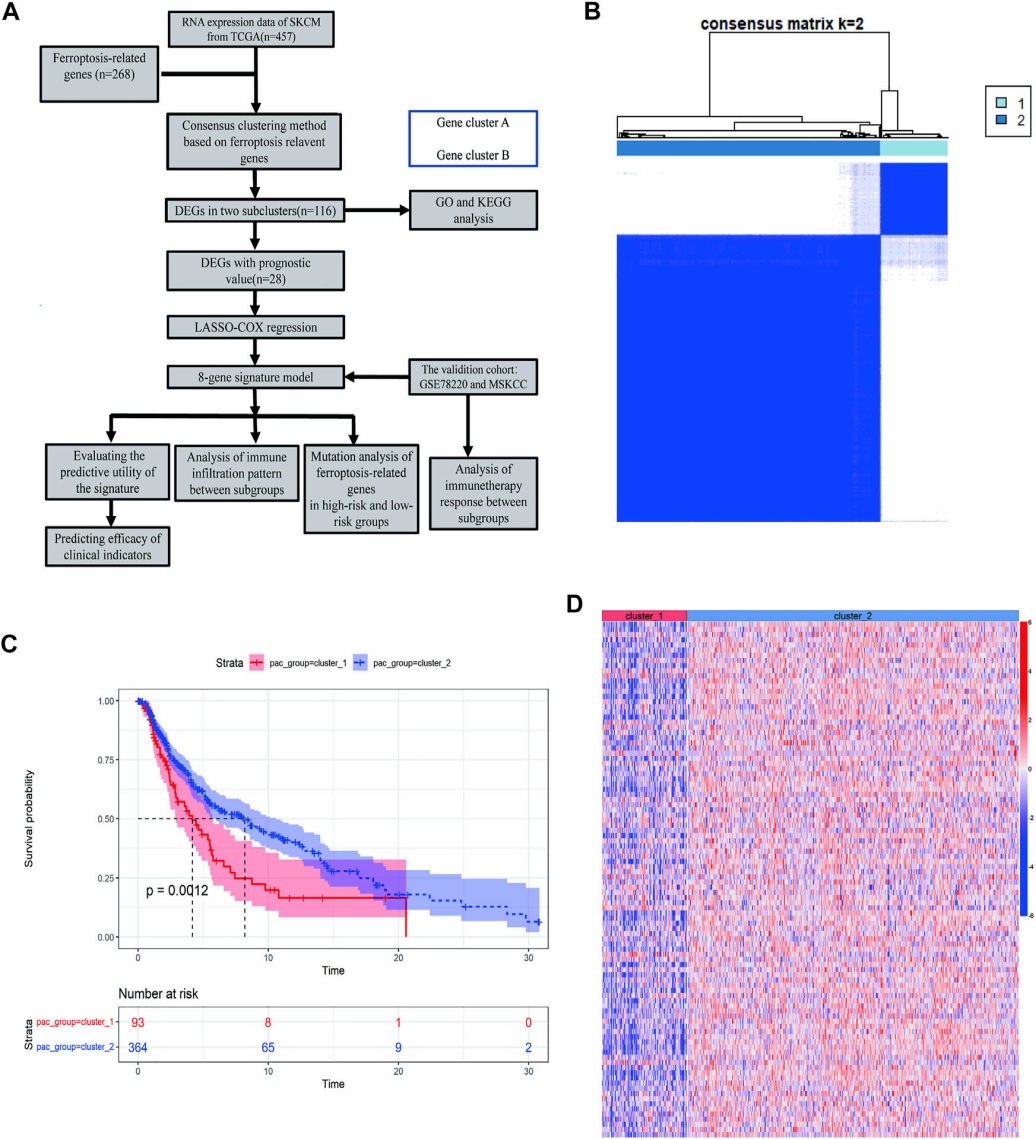
Identification of melanoma subtypes using consensus clustering in the ferroptosis set. **(A)** Flow chart of the study. **(B)** Consensus clustering applied on 268 ferroptosis-related genes. Samples were divided into cluster 1 and cluster 2. **(C)** Survival analysis of samples in Clusters 1 and 2 in TCGA cohort. **(D)** Heat map of ferroptosis-related genes expression in different clusters.

To describe our research systematically and comprehensively, a flow chart is shown in Figure 1A. After filtering out normal samples and samples without survival data, we obtained 457 gene expression profiles of SKCM samples from the TCGA dataset. From the FerrDb database and previous literature, 268 ferroptosis-related genes were identified. A total of 84 ferroptosis-related genes were selected. Consensus clustering (CC) was used to divide melanoma samples into two clusters (cluster 1 and cluster 2). In cancer research, unsupervised class discovery classifies intrinsic populations with common biological characteristics, which may exist but are unknown. The CC method is a type of unsupervised class discovery, which provides quantitative and visual stability evidence for estimating the number in a dataset (Wilkerson and Hayes, 2010). After CC, the optimal total cluster number was set to *k* = 2 (the two subclasses were designated as cluster 1 and cluster 2). When *k* = 2, the consensus matrix heat map maintained the clearest cluster partition, indicating the clustering having the highest consensus (Figure 1B; Supplementary Figure S1A–C). The *k* value determined by a cumulative density function (CDF) plot means maximum stability, at which the distribution reaches an approximate maximum (Supplementary Figure S1D). The survival curve showed a significant difference between cluster 1 and cluster 2 (Figure 1C). The gene expression heat map shows the expression of ferroptosis-related genes between the two clusters (Figure 1D).

We then analyzed the differences in the expression of ferroptosis-related genes between the two clusters and identified 116 DEGs. GO enrichment and KEGG pathway analyses were performed on the DEGs to help clarify the related biological functions and pathways of the ferroptosis-related genes. The most abundant biological processes (BP) involved include response to oxidative stress, cellular response to chemical stress, cellular response to oxidative stress, response to nutrient levels, cellular response to metal ions, ROS metabolic process, multicellular organismal homeostasis, response to reactive oxygen species, and cellular response to extracellular stimulus (Supplementary Figure S2A). In terms of molecular functions (MF), the DEGs were mainly enriched in iron ion binding and oxidoreductase activity (Supplementary Figure S2B). The main cellular component terminologies identified were protein kinase complex, autophagosome, phagophore assembly site, secondary lysosome, nucleotide-activated protein kinase complex, and promyelocytic leukemia nuclear (PML) body (Supplementary Figure S2C). Interestingly, KEGG enriched ferroptosis and some tumorigenesis-related pathways, such as melanoma (Supplementary Figure S2D). Thus, these findings indicate that the occurrence of ferroptosis is related to tumorigenesis.

### Discovering Ferroptosis-Related Genes With the Most Significant Prognostic Values

Next, the prognostic effect of ferroptosis-related genes in SKCM was examined. Among the SKCM patients in the expression matrix of the previous candidate gene set, 28 prognostic-related genes were selected through the univariate Cox regression analysis (*p* < 0.001) for further analysis (Supplementary Figure S3). There was a statistically significant difference in the expression of prognostic-related factors between the two clusters, where *ACSL1 ALOXS ARNTL CTBB FLT3 GCH1 IFNG IL33 TLR4*, and *ZEB1* were strongly significantly different (*p* ≤ 0.001) (Supplementary Figure S3A). Overall, prognostic-related factors impact on the survival and prognosis in the two clusters, especially *GCH1 CTBB TLR4 ALOX5 FLT3,* and *IFNG* (*p* < 0.0001) (Supplementary Figure S3B).

### Construction of Predictive Utility Evaluation of the Ferroptosis-Related Gene Signature

**FIGURE 2:**
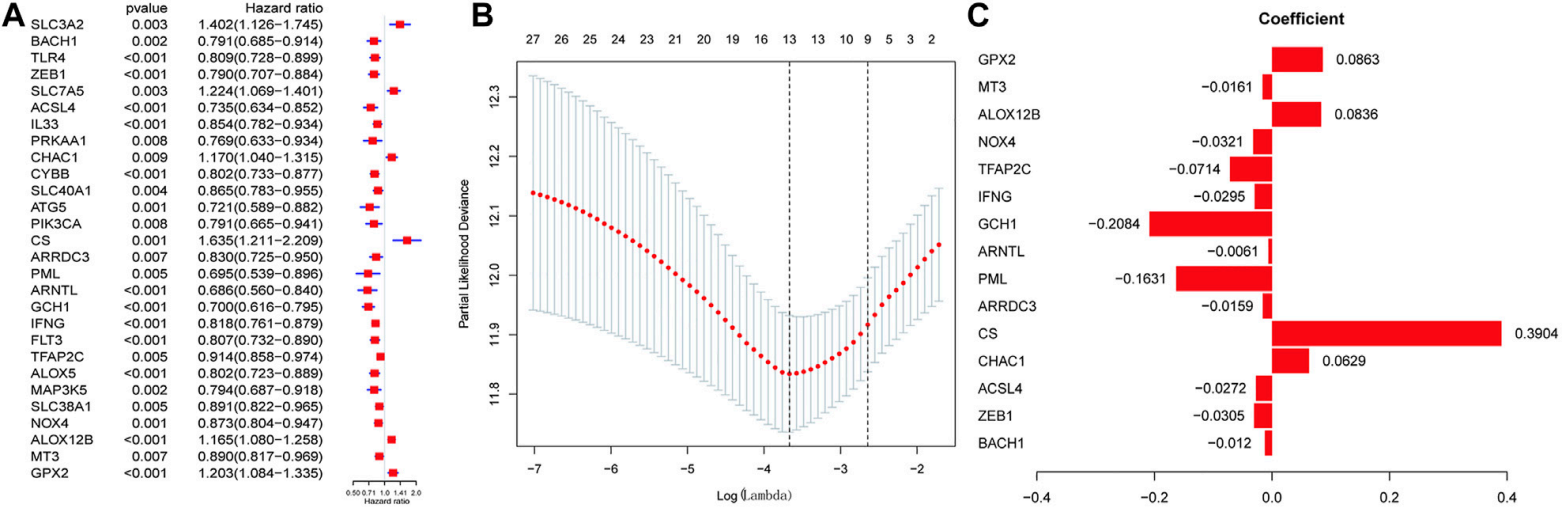
Discovering ferroptosis-related genes with the most significant prognostic values. **(A)** Univariate Cox Analysis of the candidate ferroptosis-related gene set. **(B)** Cross-validation for tuning parameter selection in the proportional hazards model. **(C)** Lasso coefficient spectrum of ferroptosis-related genes related to prognosis in SKCM.

We then performed LASSO regression analysis to establish an optimal survival-related linear risk assessment model (Figures 2A–C), which included eight genes. The purpose of LASSO regression analysis is to minimize the risk of overfitting. The risk scores of samples were calculated from regression coefficients generated by multivariate Cox regression analysis.

Through the further screening, eight genes were finally selected, where the formula for calculating the risk score was as follows: risk score = [*CHAC1* × 0.125855936071576 + *CS* × 0.560660638020698 + *PML* × (−0.30765625698674) + *GCH1* × (−0.308787668222114) + *TFAP2C* × (−0.110257841756473) + *NOX4* × (−0.06854182999606) + *ALOX12B* × 0.107841778270567 + *GPX* × 0.125495051893846]

**FIGURE 3:**
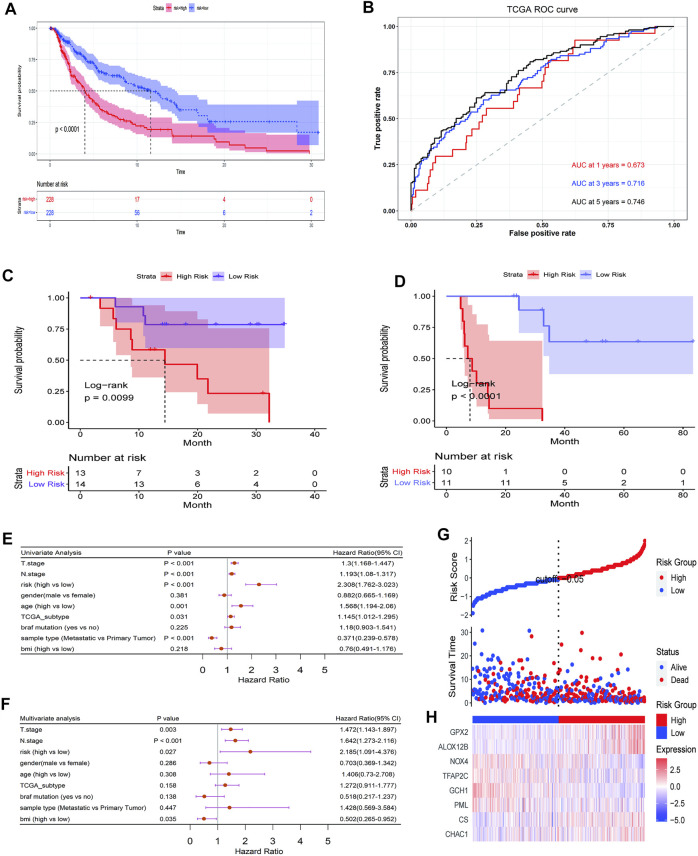
Prognostic significance of the ferroptosis-related gene signature derived risk scores in TGGA cohort. **(A)** Kaplan-Meier analysis of melanoma patients was stratified by median risk score in TCGA cohort. **(B)** Receiver operating characteristic (ROC) curve of risk score signature. **(C, D)** Kaplan-Meier analysis of melanoma patients was stratified by median risk score in GSE78220 cohort **(C)** and MSKCC cohort **(D)**. **(E, F)** Univariate and multivariate Cox regression analysis showed the predictive utility of the risk-score signature possesses excellent prognostic independence. **(G)** The curve shows the distribution of patient risk scores, survival status, and survival time. **(H)** Heatmap shows the expression of each gene in the risk-score signature.

The results obtained were used to stratify patients into low-risk (*n* = 228) and high-risk (*n* = 228) groups based on the median risk score. Clinical information for samples is shown in Table 1. We then evaluated the predictive utility of the ferroptosis-related gene signature. The K-M analysis showed that the high-risk group had a worse survival probability than the low-risk group in the TCGA cohort (Figure 3A **)**. ROC curves were used to evaluate the sensitivity of the risk model prediction, showing AUCs in the TCGA cohort for 1, 3, and 5 years, which were 0.673, 0.716, and 0.746, respectively (Figure 3B). GSE78220 and MSKCC cohorts were used to verify the validation of TGGA database risk scores. K-M survival analysis indicated that the low-risk subgroup had better overall survival (Figures 3C,D; Table 2, 3). Univariate and multivariate Cox regression analyses were performed on the TCGA cohort to test whether the eight-gene signature was a suitable independent prognostic indicator. Univariate Cox regression revealed that high-risk scores, T stage, N stage, and age were associated with poor survival prognosis (*p* < 0.001; Figure 3E). Through multivariate Cox regression, T stage, N stage, and risk score were independent predictors of melanoma (*p* < 0.05; Figure 3F). The patients were ranked from left to right according to the increasing risk scores, and a scatter plot shows the distribution of patients according to their risk scores (Figure 3G). A heat map presented differentially expressed ferroptosis-related genes between the high-risk and low-risk groups (Figure 3H).

**TABLE 1:** Characteristics of patients in low- and high-risk scores in TGGA cohort.

Characteristic	High, *N* = 228^a^	Low, *N* = 228^a^
Age	60 (51, 71)	56 (45, 68)
Gender		
Female	90 (39%)	82 (36%)
Male	138 (61%)	146 (64%)
Bmi	26.1 (23.0, 30.2)	28.0 (24.9, 33.3)
Unknown	97	121
M.stage		
M0	206 (95%)	200 (93%)
M1	10 (4.6%)	14 (6.5%)
Unknown	12	14
N.stage		
N0	117 (53%)	109 (50%)
N1	31 (14%)	42 (19.8%)
N2	23 (10.4%)	25 (11.6%)
N3	29 (13%)	27 (12%)
NX	21 (9.5%)	13 (6.0%)
Unknown	7	12
T.stage		
T0	6 (2.8%)	17 (8.1%)
T1	13 (5.9%)	27 (12.8%)
T2	37 (17%)	40 (19%)
T3	42 (19.3%)	48 (22.8%)
T4	97 (44%)	51 (24.1%)
Tis	6 (2.8%)	1 (0.5%)
TX	17 (7.8%)	27 (13%)
Unknown	10	17
TCGA_subtype		
-	12 (7.6%)	6 (3.6%)
BRAF_Hotspot_Mutants	58 (37%)	88 (53%)
NF1_Any_Mutants	13 (8.2%)	12 (7.3%)
RAS_Hotspot_Mutants	47 (30%)	44 (27%)
Triple_WT	28 (18%)	15 (9.1%)
Unknown	70	63
Sample_type		
Additional Metastatic	1 (0.4%)	0 (0%)
Metastatic	151 (66%)	206 (90%)
Primary Tumor	76 (33%)	22 (9.6%)
Braf		
False	112 (49%)	83 (36%)
True	116 (51%)	145 (64%)

a Median (IQR); *n* (%).

**TABLE 2:** Characteristics of patients in low- and high-risk scores in GSE78220 cohort.

Characteristic	High, *N* = 13^a^	Low, *N* = 14^a^
Gender		
Female	4 (31%)	4 (29%)
Male	9 (69%)	10 (71%)
Age	63 (55, 70)	58 (54, 64)
OS_time	12 (6, 20)	18 (14, 30)
OS_Status	9 (69%)	3 (21%)
ICI Response		
CR/PR	4 (31%)	10 (71%)
SD/PD	9 (69%)	4 (29%)

a n (%); Median (IQR).

**TABLE 3:** Characteristics of patients in low- and high-risk scores in MSKCC cohort.

**Characteristic**	**High, *N* = 10^a^**	**Low, *N* = 11^a^**
Age	60 (56, 63)	54 (46, 64)
Sex		
Female	6 (60%)	6 (55%)
Male	4 (40%)	5 (45%)
OS_time	8 (6, 13)	35 (28, 53)
OS_Status		
Alive	0 (0%)	8 (73%)
Dead	10 (100%)	3 (27%)
ICI Response		
CR/PR	0 (0%)	8 (73%)
SD/PD	10 (100%)	3 (27%)

a Median (IQR); *n* (%).

### Incorporating Ferroptosis Risk Scores into the Nomogram and Validation of its Clinical Benefit

**FIGURE 4:**
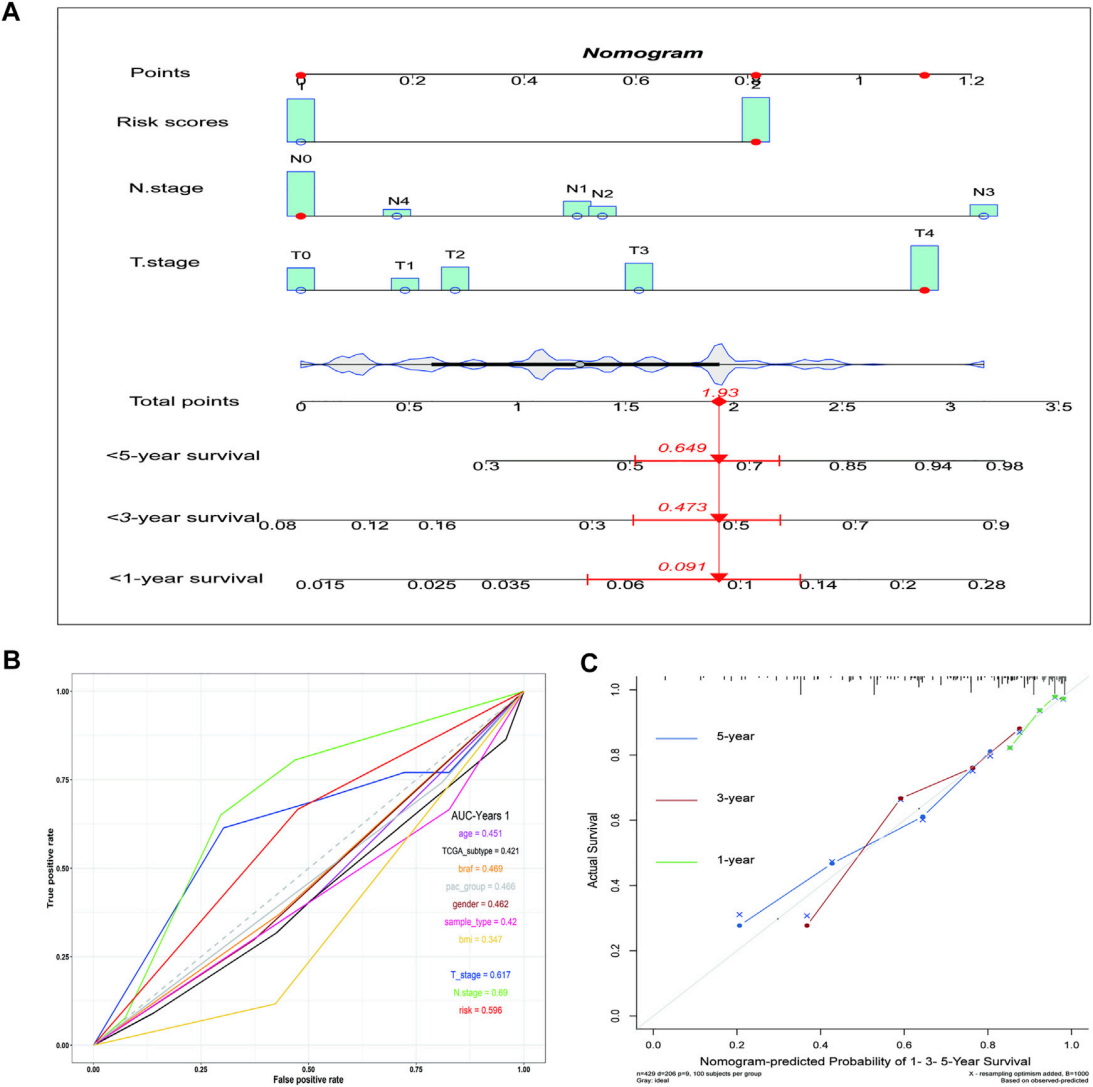
The nomogram shows the impact of various clinical features on survival prognosis in melanoma. **(A)** A nomogram of the melanoma cohort used to indicate the clinical information and overall survival. **(B)** ROC curve of clinical features. **(C)** Calibration maps used to predict the 1, 3, and 5-years survival. The *x*-axis and *y*-axis represent the expected and actual survival rates of the nomogram. The solid line represents the predicted nomogram, and the vertical line represents the 95% confidence interval.

Subsequently, a nomogram was created, which predicted the probability of specific clinical outcomes or events based on the values of multiple variables. The factors for the establishment of the nomogram included the risk scores and tumor stages. In the nomogram, columnar height represents the distribution and number of patients. For instance, for a patient in the high-risk group, the tumor stage was N0 and T4, and their total score was 1.93. The probability of their survival time being <1 year was 0.091, <3 years was 0.473, and <5 years was 0.645 (Figure 4A). The ROC curve indicates that the N-stage, T-stage, and risk scores result in better predictions than other clinical futures (Figure 4B). The calculated C index was 0.70. The calibration curve results for the 1-, 3-, and 5-years survival rates showed that the predicted survival rate was closely related to the actual rate (Figure 4C).

### Associations Between the Ferroptosis-Related Risk Scores and Immune Cell Infiltration Patterns and Immunotherapy

**FIGURE 5:**
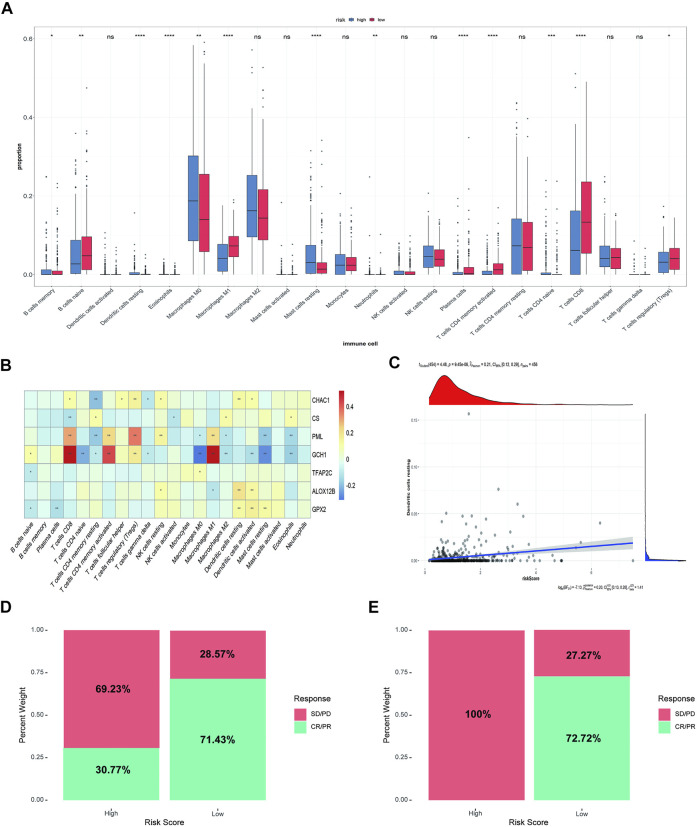
Immune correlation analysis between high-risk and low-risk groups and Comparison of rick scores and chemotherapy drug therapeutic effect. **(A)** The immune infiltration analysis between risk groups. **(B)** The correlation analysis between immune cells and 8 ferroptosis-related genes (ns: not significant, *: *p* ≤ 0.05, **: *p* ≤ 0.01; ***: *p* ≤ 0.001). **(C)** The correlation analysis between risk score and dendritic cells. **(D)** The distribution of the drug response of samples in the high-risk group and low-risk group in GSE78220 cohort. **(E)** The distribution of the drug response of samples in the high-risk group and low-risk group in MSKCC cohort.

We analyzed the difference in immune infiltration between high-risk and low-risk groups. In addition, the correlation between immune cells and model genes or risk scores were analyzed. The results showed that 12 of the 22 immune cells had significantly difference in the proportion between the high-risk and low-risk groups. Macrophages M0 and M2, mast cells, monocytes, and CD4^+^ T cells accounted for a relatively high proportion of cells in the high-risk group. In contrast, B-native cells, macrophages M1, and CD8^+^ cells accounted for a relatively high proportion of cells in the low-risk group, indicating a correlation between the prognosis difference of risk score and the immune infiltration of cancer tissue (Figure 5A). Subsequently, the correlation between immune cells and model genes was analyzed. The results showed that the expression of *GCH1* significantly correlated with macrophage M1 cells, CD8^+^ T cells, and activated CD4^+^ memory T cells (Figure 5B). The correlation analysis between immune cell infiltration patterns and risk scores showed a specific correlation between risk scores and dendritic cells (Figure 5C).

Then, we analyzed the relationship between risk score and response to immunotherapy in the external validation cohorts, to further evaluate the effect of immunotherapy further. In both independent cohorts, complete response (CR) or partial response (PR) to immunotherapy drugs accounted for more samples in the low-risk group than in the high-risk group. Almost all the samples in the high-risk group responded to PD-1 chemotherapeutic drugs with progressive disease (PD)/stable disease (SD), especially in the MSKCC cohort, indicating that the low-risk group received more benefits in terms of the risk score than the high-risk group (Figures 5D,E).

### The Relationship Between Ferroptosis-Related Risk Scores and Somatic Mutations

**FIGURE 6:**
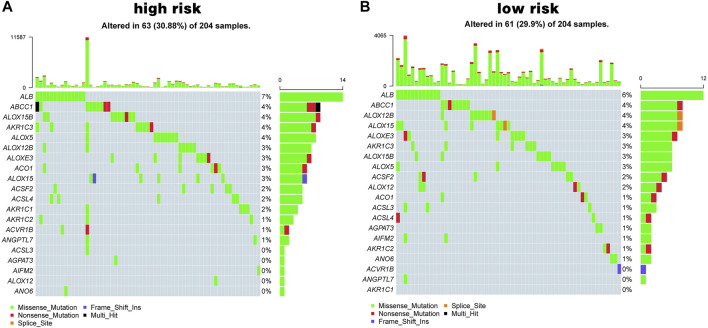
Ferroptosis mutation map. **(A, B)** The top 20 mutated genes in high-risk group **(A)** and in low-risk group **(B)**.

In addition, mutational differences in the ferroptosis-related genes between the high-risk group and low-risk group were analyzed. Figure 6 shows the top 20 genes in terms of ferroptosis-related gene mutations; *ALB* and *ABCC1* had the highest mutation rates in both the high- and low-risk groups.

## Discussion

Cell death depends on special regulated molecular mechanisms regulation called regulated cell death (RCD) (Galluzzi et al., 2018). Modern medical researches uses these unique biological processes to treat cancer by allowing cancer cells to die, relying on dedicated molecular machinery pharmacologically or genetically. Caspase-dependent apoptosis is a generally recognized RCD process against cancer (Liang et al., 2019). However, tumor cells have the characteristics of resistance to apoptosis, and drug resistance will also occur in the process of chemotherapy-induced apoptosis of cancer (Gottesman et al., 2002). Therefore, it is necessary to find some new forms of RCD to develop anticancer drugs.

Ferroptosis is an iron-dependent form of RCD characterized by the overwhelming accumulation of lethal lipid peroxidation; it is different from apoptosis, necrosis, and autophagy (Dixon et al., 2012). Ferroptosis can be triggered by exogenous small molecules (such as erastin, sorafenib, or sulfasalazine) or regulating physiological conditions (such as the high extracellular glutamate concentration) to block the X_C_^−^ system. Other ferroptosis inducers can directly inhibit GPX4, ultimately leading to lipid peroxide accumulation (Liang et al., 2019). Increasing oxidizable polyunsaturated phospholipids or interfering with the iron balance to destroy the balance of lipid metabolism balance can also sensitize cells to ferroptosis, Strong iron dependence can make cancer cells more susceptible to iron overload and ROS accumulation, enabling tumor microenvironment-targeted, ferroptosis-mediated cancer therapy (Vigil et al., 2010; Hassannia et al., 2019). Studies have found that clear cell carcinomas, highly aggressive malignancies, are usually not susceptible to conventional anticancer treatment. However, their unique metabolic state has been identified to be susceptible to ferroptosis (Zou et al., 2019). In addition, a large number of studies have confirmed the crucial role of ferroptosis in inducing cancer cells death and inhibiting tumor growth. The metabolic status of breast cancer, liver cancer, colorectal cancer, and other malignant tumors is closely related to ferroptosis (Sun et al., 2015). However, most current studies focus on the role of iron metabolism in cancer occurrence and treatment, and the relationship between genes related to ferroptosis and cancer prognosis remains to be explored.

Melanoma cells have a highly mutagenic nature and an immune escape mechanism (Davis et al., 2019), including downregulation of the expression of tumor-associated antigens and melanoma differentiation antigens to inhibit cytotoxic T cell recognition and clearance of tumor cells and secretion of immune inhibitory molecules such as transforming growth factor-beta (TGF-β) and prostaglandin E2 to escape immunity (Palmer et al., 2011; Pitcovski et al., 2017). In addition, melanoma can close the immune response by expressing programmed cell death protein 1/2 (PD-1/2) to avoid immune destruction (Passarelli et al., 2017). So far, the most effective treatment for metastatic melanoma is immune checkpoint inhibitors, such as anti-PD1, PD-L1/2, and CTLA4 antibodies. Still, the complications of these treatments are serious, and many of patients have inadequate treatment responses. So, the combination of ferroptosis and immunotherapy may have good prospects for melanoma therapy. Studies have found that immunotherapy activated CD8^+^ T cells enhance iron-specific lipid peroxidation in tumor cells, and increased ferroptosis contributes to the antitumor effect of immunotherapy (Wang et al., 2019, 8). At present, the relationship between ferroptosis and tumor immunotherapy is still not very clear. Nevertheless, the establishment of some prognostic signatures can be established that use the ferroptosis-related genes to evaluate different tumor immune characteristics to guide individualized immunotherapy (Zhuo et al., 2020; Jiang et al., 2021).

This study found that the ferroptosis-related genes can classify melanoma patients into two classes that exhibit significant differences in clinical and molecular features. Patients were classified into high-risk and low-risk groups by LASSO regression analysis. We established a prognostic model based on eight genes, which were composed of the risk-related genes (*CHAC1*, *CS*, *GPX2*, and *ALOX12B*) and the protective genes *(PML*, *GCH1*, *TFAP2C*, and *NOX4*). The GCH1-BH4 pathway is a new pathway that is independent of the GPX4/glutathione system and regulates ferroptosis. GCH1 is a rate-limiting enzyme for the synthesis of tetrahydrobiopterin. Overexpression of *GCH1* can eliminate lipid peroxidation and almost completely inhibit ferroptosis (Wei et al., 2020). In high-grade serous ovarian cancers, due to the silencing of *PML*, ROS content, lipid peroxidation, and lysosomes, and the lysosomal Fe2^+^levels are reduced, which can result in potential ferroptosis and improved sensitivity to immunotherapy (Gentric et al., 2019). *NOX4* encodes ROS-producing enzymes enriched in the kidney, where high expression is an essential source of renal ROS. Meanwhile, inhibition of *NOX4* reduces the cystine deprivation-induced cell death and lipid ROS, suggesting its vital role in ferroptosis (Yang et al., 2019). *TFAP2C* plays an essential role in cell differentiation, tissue development, and tumor biology. *TFAP2C* upregulates the *GPX4* gene in tumor cells and regulates some ferroptosis regulators, such as epidermal growth factor receptor (EGFR), CDKN1A, and YAP1, thereby negatively regulating ferroptosis (Dai et al., 2020). Mitochondria play a central role in fatty acid metabolism and provide specific lipid precursors for lipid oxidation. *CS* participates in mitochondrial lipid metabolism and regulates the activation and synthesis of fatty acids. Silencing *CS* can rescue cell viability from erastin-induced ferroptosis (Wang et al., 2020). *ALOX12B* encodes lipoxygenase, which is associated with autosomal dominant fish scale disease and proliferation of epidermoid carcinoma cells (Jiang et al., 2020). Glutathione-specific g-glutamyl cyclotransferase (*CHAC1*) is a downstream target of the eIF2alpha-*ATF4* pathway, and *CHAC1* upregulation may be useful as a Pharmacodynamic marker for cystine or cysteine-starved cells (Dixon et al., 2014). Overexpression of *CHAC1* led to a robust depletion of glutathione, which was alleviated in a CHAC1 catalytic mutant. On activating the expression of *ATF4* and *CHAC1,* the initial glutathione depletion by inhibiting cystine transport leads to ferroptosis (Ratan, 2020). In summary, numerous studies have shown that the above genes are related to ferroptosis, providing theoretical support for our risk model.

After establishing the ferroptosis-related risk model, our samples were divided into high-risk and low-risk groups according to the median risk score. We used statistical methods such as univariate and multivariate Cox regression analysis to show that the risk score model is an excellent independent prognostic indicator for evaluating the overall survival of melanoma patients. Subsequently, we developed a nomogram, combined with multiple clinical indicators and biological attributes, set up a personalized prognostic prediction scale, and quantified the risk of different individuals with numerous risk factors. In addition, we also analyzed the immune correlation between high- and low-risk groups and found that the risk score could indicate the relationship between prognosis difference and immune tissue infiltration, which could better guide the immunotherapy of SKCM.

Finally, our research also has certain limitations. As a retrospective study, it cannot fully contain all clinical data, so there will be some limitations in variable selection, which must be verified by selecting as many cohorts as possible. In addition, there should be prospective studies to further evaluate the clinical value of our model. Finally, a series of experiments should be carried out to explore the further evaluate the prognostic value of the eight ferroptosis-related gene signature.

## Data Availability

The datasets presented in this study can be found in online repositories. The names of the repository/repositories and accession number(s) can be found in the article/Supplementary Material.

## References

[B1] Aris M. Barrio M. a. M. (2015). Combining Immunotherapy with Oncogene-Targeted Therapy: A New Road for Melanoma Treatment. Front. Immunol. 6. 10.3389/fimmu.2015.00046 PMC4321613 25709607

[B2] Battaglia A. M. Chirillo R. Aversa I. Sacco A. Costanzo F. Biamonte F. (2020). Ferroptosis and Cancer: Mitochondria Meet the “Iron Maiden” Cell Death. Cells 9, 1505. 10.3390/cells9061505 PMC7349567 32575749

[B3] Bogdan A. R. Miyazawa M. Hashimoto K. Tsuji Y. (2016). Regulators of Iron Homeostasis: New Players in Metabolism, Cell Death, and Disease. Trends Biochem. Sci. 41, 274–286. 10.1016/j.tibs.2015.11.012 26725301 PMC4783254

[B4] Bridges R. J. Natale N. R. Patel S. A. (2012). System Xc- Cystine/glutamate Antiporter: an Update on Molecular Pharmacology and Roles within the CNS. Br. J. Pharmacol. 165, 20–34. 10.1111/j.1476-5381.2011.01480.x 21564084 PMC3252963

[B5] Coit D. G. Andtbacka R. Anker C. J. Bichakjian C. K. Carson W. E. Daud A. (2013). Melanoma, Version 2.2013. J. Natl. Compr. Canc Netw. 11, 395–407. 10.6004/jnccn.2013.0055 23584343

[B6] Costa da Silva M. Breckwoldt M. O. Vinchi F. Correia M. P. Stojanovic A. Thielmann C. M. (2017). Iron Induces Anti-tumor Activity in Tumor-Associated Macrophages. Front. Immunol. 8, 1479. 10.3389/fimmu.2017.01479 29167669 PMC5682327

[B7] Dai C. Chen X. Li J. Comish P. Kang R. Tang D. (2020). Transcription Factors in Ferroptotic Cell Death. Cancer Gene Ther. 27, 645–656. 10.1038/s41417-020-0170-2 32123318

[B8] Davis L. E. Shalin S. C. Tackett A. J. (2019). Current State of Melanoma Diagnosis and Treatment. Cancer Biol. Ther. 20, 1366–1379. 10.1080/15384047.2019.1640032 31366280 PMC6804807

[B9] Dixon S. J. Lemberg K. M. Lamprecht M. R. Skouta R. Zaitsev E. M. Gleason C. E. (2012). Ferroptosis: an Iron-dependent Form of Nonapoptotic Cell Death. Cell 149, 1060–1072. 10.1016/j.cell.2012.03.042 22632970 PMC3367386

[B10] Dixon S. J. Patel D. N. Welsch M. Skouta R. Lee E. D. Hayano M. (2014). Pharmacological Inhibition of Cystine-Glutamate Exchange Induces Endoplasmic Reticulum Stress and Ferroptosis. eLife 3, e02523. 10.7554/eLife.02523 24844246 PMC4054777

[B11] Fearnhead H. O. Vandenabeele P. Vanden Berghe T. (2017). How Do We Fit Ferroptosis in the Family of Regulated Cell Death? Cell Death Differ 24, 1991–1998. 10.1038/cdd.2017.149 28984871 PMC5686356

[B12] Galluzzi L. Vitale I. Aaronson S. A. Abrams J. M. Adam D. Agostinis P. (2018). Molecular Mechanisms of Cell Death: Recommendations of the Nomenclature Committee on Cell Death 2018. Cel Death Differ 25, 486–541. 10.1038/s41418-017-0012-4 PMC5864239 29362479

[B13] Gentric G. Kieffer Y. Mieulet V. Goundiam O. Bonneau C. Nemati F. (2019). PML-regulated Mitochondrial Metabolism Enhances Chemosensitivity in Human Ovarian Cancers. Cel Metab. 29, 156–173. 10.1016/j.cmet.2018.09.002 PMC6331342 30244973

[B14] Gottesman M. M. Fojo T. Bates S. E. (2002). Multidrug Resistance in Cancer: Role of ATP-dependent Transporters. Nat. Rev. Cancer 2, 48–58. 10.1038/nrc706 11902585

[B15] Hassannia B. Vandenabeele P. Vanden Berghe T. (2019). Targeting Ferroptosis to Iron Out Cancer. Cancer Cell 35, 830–849. 10.1016/j.ccell.2019.04.002 31105042

[B16] Jiang P. Yang F. Zou C. Bao T. Wu M. Yang D. (2021). The Construction and Analysis of a Ferroptosis-Related Gene Prognostic Signature for Pancreatic Cancer. Aging 13, 10396–10414. 10.18632/aging.202801 33819918 PMC8064155

[B17] Jiang T. Zhou B. Li Y. Yang Q. Tu K. Li L. (2020). ALOX12B Promotes Carcinogenesis in Cervical Cancer by Regulating the PI3K/ERK1 Signaling Pathway. Oncol. Lett. 20, 1360–1368. 10.3892/ol.2020.11641 32724378 PMC7377187

[B18] Leonardi G. Candido S. Falzone L. Spandidos D. Libra M. (2020). Cutaneous Melanoma and the Immunotherapy Revolution (Review). Int. J. Oncol. 57, 609–618. 10.3892/ijo.2020.5088 32582963 PMC7384846

[B19] Liang C. Zhang X. Yang M. Dong X. (2019). Recent Progress in Ferroptosis Inducers for Cancer Therapy. Adv. Mater. 31, 1904197. 10.1002/adma.201904197 31595562

[B20] Liang J.-Y. Wang D.-S. Lin H.-C. Chen X.-X. Yang H. Zheng Y. (2020). A Novel Ferroptosis-Related Gene Signature for Overall Survival Prediction in Patients with Hepatocellular Carcinoma. Int. J. Biol. Sci. 16, 2430–2441. 10.7150/ijbs.45050 32760210 PMC7378635

[B21] Luo M. Wu L. Zhang K. Wang H. Zhang T. Gutierrez L. (2018). miR-137 Regulates Ferroptosis by Targeting Glutamine Transporter SLC1A5 in Melanoma. Cel Death Differ 25, 1457–1472. 10.1038/s41418-017-0053-8 PMC6113319 29348676

[B22] Mou Y. Wang J. Wu J. He D. Zhang C. Duan C. (2019). Ferroptosis, a New Form of Cell Death: Opportunities and Challenges in Cancer. J. Hematol. Oncol. 12, 34. 10.1186/s13045-019-0720-y 30925886 PMC6441206

[B23] Newman A. M. Liu C. L. Green M. R. Gentles A. J. Feng W. Xu Y. (2015). Robust Enumeration of Cell Subsets from Tissue Expression Profiles. Nat. Methods 12, 453–457. 10.1038/nmeth.3337 25822800 PMC4739640

[B24] Palmer S. R. Erickson L. A. Ichetovkin I. Knauer D. J. Markovic S. N. (2011). Circulating Serologic and Molecular Biomarkers in Malignant Melanoma. Mayo Clinic Proc. 86, 981–990. 10.4065/mcp.2011.0287 PMC3184027 21964175

[B25] Passarelli A. Mannavola F. Stucci L. S. Tucci M. Silvestris F. (2017). Immune System and Melanoma Biology: a Balance between Immunosurveillance and Immune Escape. Oncotarget 8, 106132–106142. 10.18632/oncotarget.22190 29285320 PMC5739707

[B26] Pitcovski J. Shahar E. Aizenshtein E. Gorodetsky R. (2017). Melanoma Antigens and Related Immunological Markers. Crit. Rev. Oncology/Hematology 115, 36–49. 10.1016/j.critrevonc.2017.05.001 28602168

[B27] Ratan R. R. (2020). The Chemical Biology of Ferroptosis in the Central Nervous System. Cel Chem. Biol. 27, 479–498. 10.1016/j.chembiol.2020.03.007 PMC7245561 32243811

[B28] Rodríguez-Cerdeira C. Carnero Gregorio M. López-Barcenas A. Sánchez-Blanco E. Sánchez-Blanco B. Fabbrocini G. (20172017). Advances in Immunotherapy for Melanoma: A Comprehensive Review. Mediators Inflamm. 2017, 1–14. 10.1155/2017/3264217 PMC5564072 28848246

[B29] Sacco A. Battaglia A. M. Botta C. Aversa I. Mancuso S. Costanzo F. (2021). Iron Metabolism in the Tumor Microenvironment-Implications for Anti-cancer Immune Response. Cells 10, 303. 10.3390/cells10020303 33540645 PMC7913036

[B30] Sato H. Tamba M. Ishii T. Bannai S. (1999). Cloning and Expression of a Plasma Membrane Cystine/glutamate Exchange Transporter Composed of Two Distinct Proteins. J. Biol. Chem. 274, 11455–11458. 10.1074/jbc.274.17.11455 10206947

[B31] Snyder A. Makarov V. Merghoub T. Yuan J. Zaretsky J. M. Desrichard A. (2014). Genetic Basis for Clinical Response to CTLA-4 Blockade in Melanoma. N. Engl. J. Med. 371, 2189–2199. 10.1056/NEJMoa1406498 25409260 PMC4315319

[B32] Sun X. Ou Z. Xie M. Kang R. Fan Y. Niu X. (2015). HSPB1 as a Novel Regulator of Ferroptotic Cancer Cell Death. Oncogene 34, 5617–5625. 10.1038/onc.2015.32 25728673 PMC4640181

[B33] Tang R. Xu J. Zhang B. Liu J. Liang C. Hua J. (2020). Ferroptosis, Necroptosis, and Pyroptosis in Anticancer Immunity. J. Hematol. Oncol. 13, 110. 10.1186/s13045-020-00946-7 32778143 PMC7418434

[B34] Ursini F. Maiorino M. Brigelius-Flohé R. Aumann K. D. Roveri A. Schomburg D. (1995). Diversity of Glutathione Peroxidases. Methods Enzymol. 252, 38–53. 10.1016/0076-6879(95)52007-4 7476373

[B35] Vigil D. Cherfils J. Rossman K. L. Der C. J. (2010). Ras Superfamily GEFs and GAPs: Validated and Tractable Targets for Cancer Therapy? Nat. Rev. Cancer 10, 842–857. 10.1038/nrc2960 21102635 PMC3124093

[B36] Wang H. Liu C. Zhao Y. Gao G. (2020). Mitochondria Regulation in Ferroptosis. Eur. J. Cel Biol. 99, 151058. 10.1016/j.ejcb.2019.151058 31810634

[B37] Wang W. Green M. Choi J. E. Gijón M. Kennedy P. D. Johnson J. K. (2019). CD8+ T Cells Regulate Tumour Ferroptosis during Cancer Immunotherapy. Nature 569, 270–274. 10.1038/s41586-019-1170-y 31043744 PMC6533917

[B38] Wei X. Yi X. Zhu X.-H. Jiang D.-S. (20202020). Posttranslational Modifications in Ferroptosis. Oxidative Med. Cell Longevity 2020, 1–12. 10.1155/2020/8832043 PMC7718049 33294126

[B39] Welch H. G. Mazer B. L. Adamson A. S. (2021). The Rapid Rise in Cutaneous Melanoma Diagnoses. N. Engl. J. Med. 8. 10.1056/nejmsb2019760 33406334

[B40] Wilkerson M. D. Hayes D. N. (2010). ConsensusClusterPlus: a Class Discovery Tool with Confidence Assessments and Item Tracking. Bioinformatics 26, 1572–1573. 10.1093/bioinformatics/btq170 20427518 PMC2881355

[B41] Yang W.-H. Ding C.-K. C. Sun T. Rupprecht G. Lin C.-C. Hsu D. (2019). The Hippo Pathway Effector TAZ Regulates Ferroptosis in Renal Cell Carcinoma. Cel Rep. 28, 2501–2508. e4. 10.1016/j.celrep.2019.07.107 PMC10440760 31484063

[B42] Yang W. S. SriRamaratnam R. Welsch M. E. Shimada K. Skouta R. Viswanathan V. S. (2014). Regulation of Ferroptotic Cancer Cell Death by GPX4. Cell 156, 317–331. 10.1016/j.cell.2013.12.010 24439385 PMC4076414

[B43] Yu H. Guo P. Xie X. Wang Y. Chen G. (2017). Ferroptosis, a New Form of Cell Death, and its Relationships with Tumourous Diseases. J. Cel. Mol. Med. 21, 648–657. 10.1111/jcmm.13008 PMC5345622 27860262

[B44] Zhang K. Wu L. Zhang P. Luo M. Du J. Gao T. (2018). miR‐9 Regulates Ferroptosis by Targeting Glutamic‐oxaloacetic Transaminase GOT1 in Melanoma. Mol. Carcinogenesis 57, 1566–1576. 10.1002/mc.22878 30035324

[B45] Zhuo S. Chen Z. Yang Y. Zhang J. Tang J. Yang K. (2020). Clinical and Biological Significances of a Ferroptosis-Related Gene Signature in Glioma. Front. Oncol. 10, 590861. 10.3389/fonc.2020.590861 33330074 PMC7718027

[B46] Zou Y. Palte M. J. Deik A. A. Li H. Eaton J. K. Wang W. (2019). A GPX4-dependent Cancer Cell State Underlies the clear-cell Morphology and Confers Sensitivity to Ferroptosis. Nat. Commun. 10, 1617. 10.1038/s41467-019-09277-9 30962421 PMC6453886

