# Supplementary material for: Ferroptosis-Related Gene Signature Predicts the Prognosis of Skin Cutaneous Melanoma and Response to Immunotherapy
Source: Front Genet. 2021 Nov 3;12:758981. doi: 10.3389/fgene.2021.758981 (PMC8595480; doi:10.3389/fgene.2021.758981)
Supplement: Supplementary file 1 [file DataSheet1.docx]

Supplementary Material


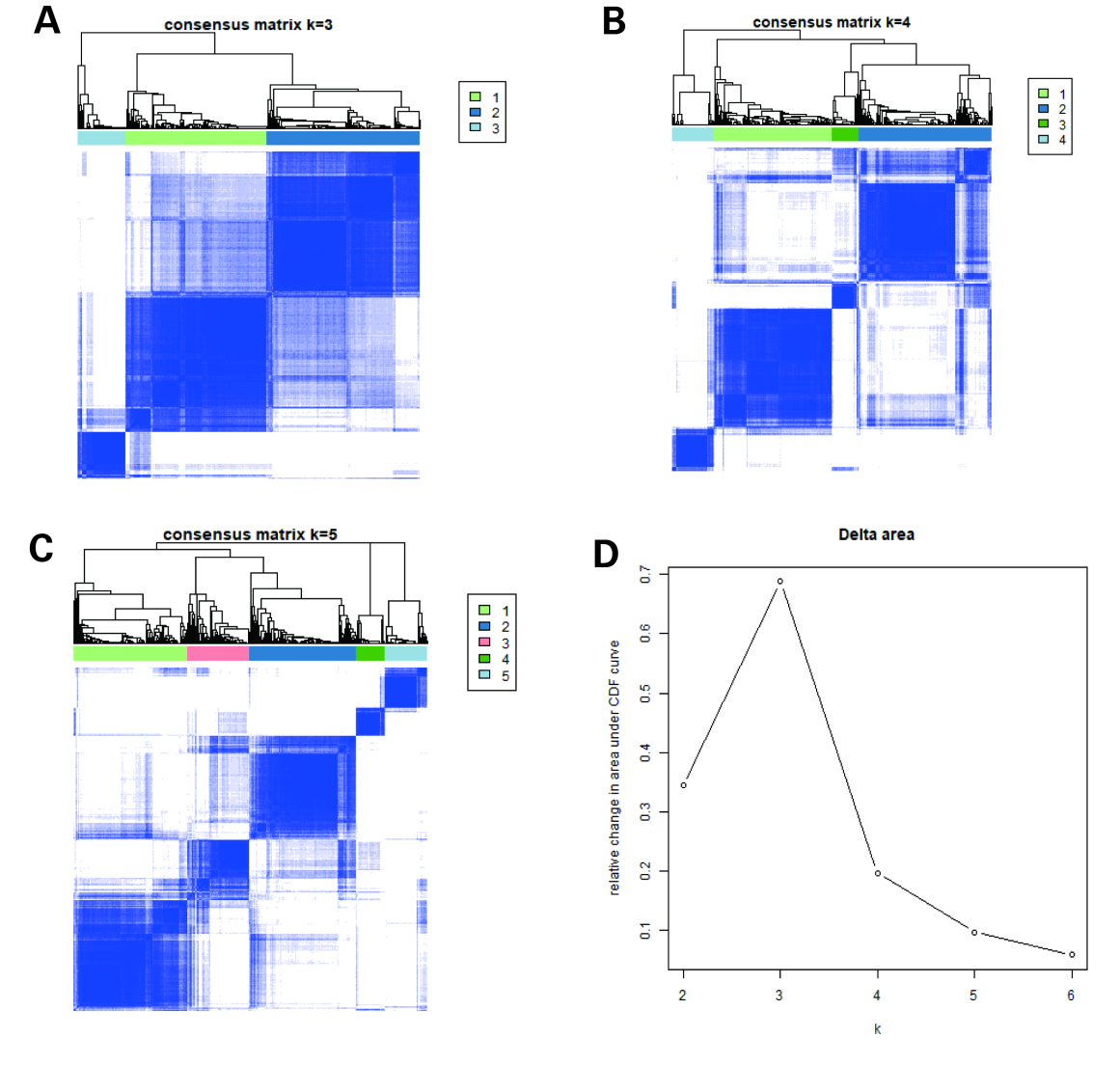


**Supplementary Figure 1.** Consensus matrix of consensus clustering for K=3(A), 4(B), 5(C) in TCGA cohort. The CDF plot means maximum stability, at which the distribution reaches an approximate maximum(D).


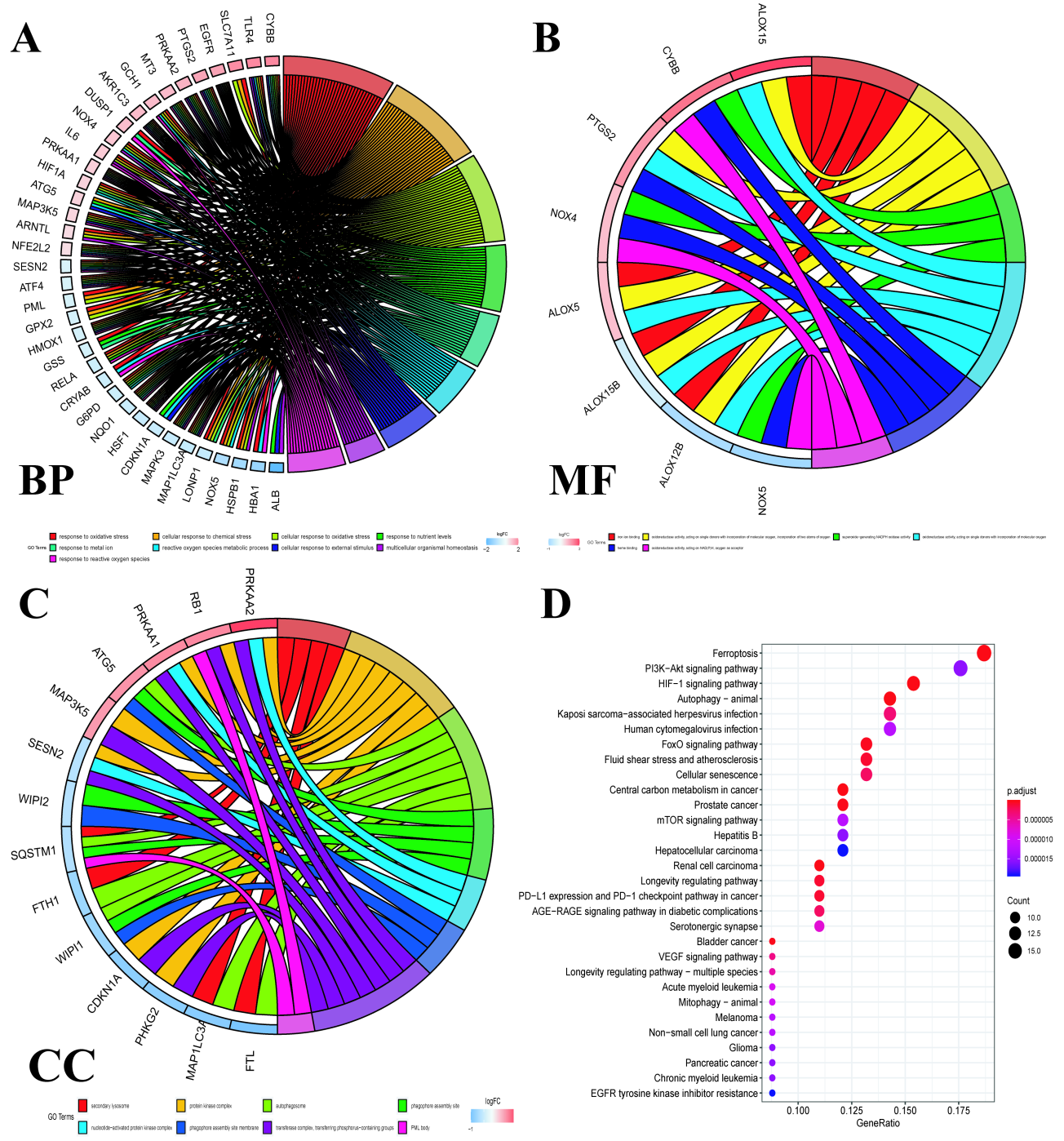


**Supplementary Figure 2** Pathway annotation of differential ferroptosis-related gene set using GO (A-C) and KEGG methods (D). “BP” represents “biological process”, “CC” represents “cellular component”, and “MF” represents “molecular function”


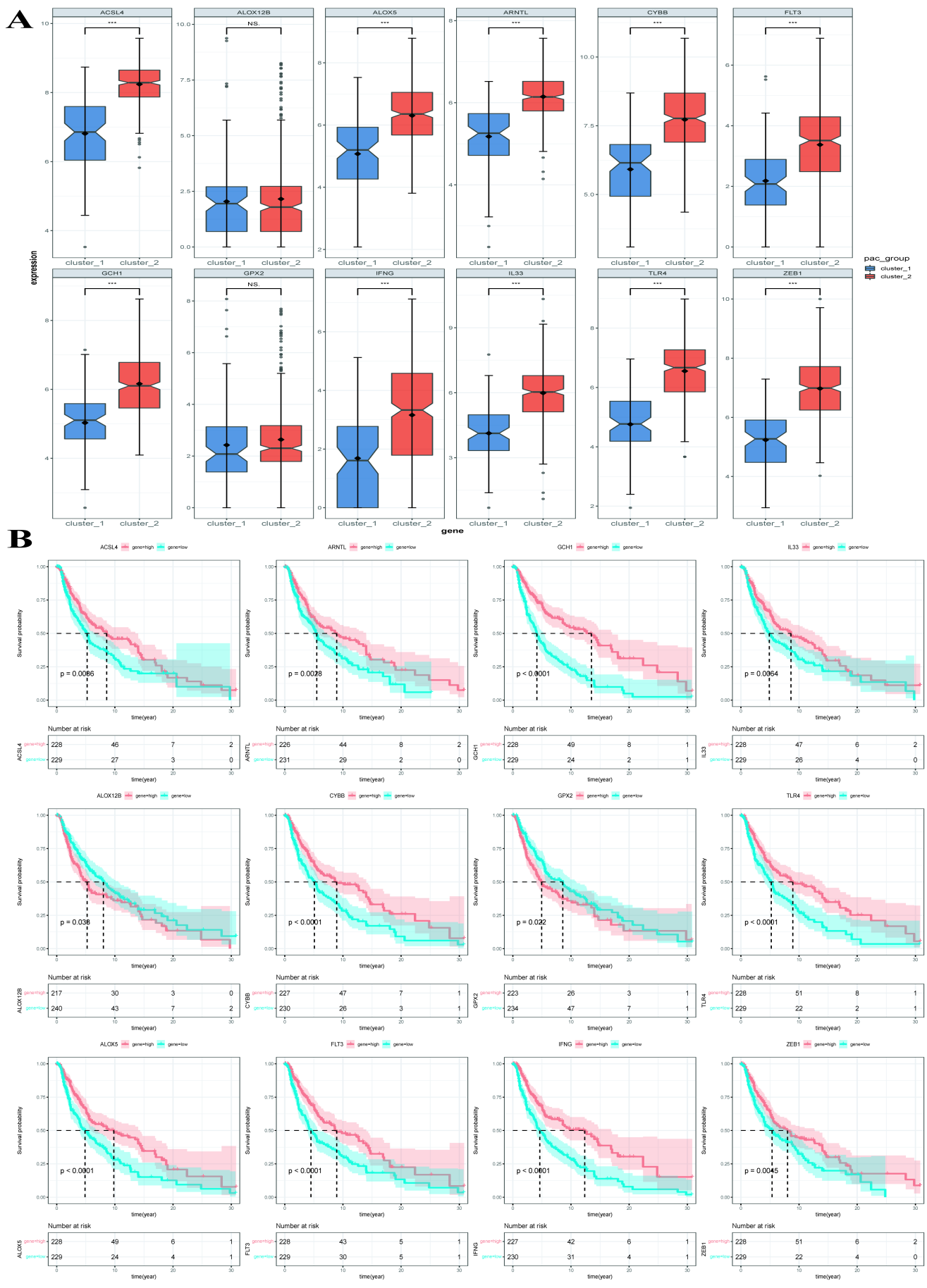


**Supplementary Figure 3.** Univariate COX regression to identify overall prognostic-related genes. The expression of genes in two subclusters (NS: not significant, ***: P ≤ 0.001) (A). The K-M survival analysis of corresponding genes (B).

# Supplementary Table

**Table S1**.the 268 ferroptosis related genes were included in this study.

| ABCC1 | CARS1 | GABARAPL2 | LPIN1 | PGD | SRXN1 |
| --- | --- | --- | --- | --- | --- |
| ACO1 | CAV1 | GABPB1 | LURAP1L | PHKG2 | STAT3 |
| ACSF2 | CBS | GCH1 | MAFG | PIK3CA | STEAP3 |
| ACSL3 | CD44 | GCLC | MAP1LC3A | PLIN2 | STMN1 |
| ACSL4 | CDKN1A | GDF15 | MAP3K5 | PLIN4 | TAZ |
| ACVR1B | CDKN2A | GLS2 | MAPK1 | PML | TF |
| AGPAT3 | CDO1 | GLUT13 | MAPK14 | PRDX1 | TFAP2C |
| AIFM2 | CEBPG | GOT1 | MAPK3 | PRDX6 | TFR2 |
| AKR1C1 | CHAC1 | GPT2 | MAPK8 | PRKAA1 | TFRC |
| AKR1C2 | CHMP5 | GPX2 | MAPK9 | PRKAA2 | TGFBR1 |
| AKR1C3 | CHMP6 | GPX4 | MIOX | PROM2 | TLR4 |
| ALB | CISD1 | HAMP | MIR137 | PSAT1 | TMBIM4 |
| ALOX12 | CISD2 | HBA1 | MIR17 | PTGS2 | TNFAIP3 |
| ALOX12B | CS | HELLS | MIR212 | RB1 | TP53 |
| ALOX15 | CXCL2 | HERPUD1 | MIR30B | RELA | TP63 |
| ALOX15B | CYBB | HIC1 | MIR4715 | RGS4 | TRIB3 |
| ALOX5 | DDIT3 | HIF1A | MIR6852 | RIPK1 | TSC22D3 |
| ALOXE3 | DDIT4 | HILPDA | MIR9-1 | RPL8 | TUBE1 |
| ANGPTL7 | DNAJB6 | HMGB1 | MIR9-2 | RRM2 | TXNIP |
| ANO6 | DPP4 | HMOX1 | MIR9-3 | SAT1 | TXNRD1 |
| ARNTL | DRD4 | HNF4A | MT1G | SCD | UBC |
| ARRDC3 | DRD5 | HRAS | MT3 | SCP2 | ULK1 |
| ASNS | DUOX1 | HSD17B11 | MTDH | SELENOS | ULK2 |
| ATF3 | DUOX2 | HSF1 | MTOR | SESN2 | VDAC2 |
| ATF4 | DUSP1 | HSPA5 | MUC1 | SETD1B | VEGFA |
| ATG13 | EGFR | HSPB1 | MYB | SIRT1 | VLDLR |
| ATG16L1 | EGLN2 | IDH1 | NCF2 | SLC1A4 | WIPI1 |
| ATG3 | EIF2AK4 | IFNG | NCOA4 | SLC1A5 | WIPI2 |
| ATG4D | EIF2S1 | IL33 | NF2 | SLC2A1 | XBP1 |
| ATG5 | ELAVL1 | IL6 | NFE2L2 | SLC2A12 | YWHAE |
| ATG7 | EMC2 | IREB2 | NFS1 | SLC2A14 | YY1AP1 |
| ATM | ENPP2 | ISCU | NGB | SLC2A3 | ZEB1 |
| ATP5MC3 | EPAS1 | JDP2 | NNMT | SLC2A6 | ZFP36 |
| ATP6V1G2 | FADS2 | JUN | NOS2 | SLC2A8 | ZFP69B |
| AURKA | FANCD2 | KEAP1 | NOX1 | SLC38A1 | ZNF419 |
| BACH1 | FBXW7 | KIM-1 | NOX3 | SLC3A2 | CARS |
| BAP1 | Fer1HCH | KLHL24 | NOX4 | SLC40A1 | GCLM |
| BECN1 | FH | KRAS | NOX5 | SLC7A11 | GSS |
| BID | FLT3 | LAMP2 | NQO1 | SLC7A5 | HMGCR |
| BLOC1S5-TXNDC5 | FTH1 | LINC00336 | NRAS | SNORA16A | CRYAB |
| BNIP3 | FTL | LINC00472 | OTUB1 | SNX4 | FDFT1 |
| BRD4 | FTMT | LOC284561 | OXSR1 | SOCS1 | HSBP1 |
| CA9 | G6PD | LOC390705 | PANX1 | SP1 | ACACA |
| CAPG | G6PDX | LONP1 | PCK2 | SQSTM1 | SQLE |
| CARS1 | GABARAPL1 | LPCAT3 | PEBP1 | SRC |  |
